# New Primary Malignancy Masquerading as Metastatic Prostate Adenocarcinoma

**DOI:** 10.1155/2015/358572

**Published:** 2015-02-19

**Authors:** Ellen A. Szwed, Sarunas Sliesoraitis, Thu-Cuc Nguyen, Minh-Nguyet Nguyen, Jan S. Moreb, Robert A. Zlotecki, Paul L. Crispen, Nam H. Dang, Long H. Dang

**Affiliations:** ^1^Division of Hematology and Oncology, Department of Internal Medicine, University of Florida Shands Cancer Center, 1600 SW Archer Road, Gainesville, FL 32610, USA; ^2^Department of Radiation Oncology, University of Florida Shands Cancer Center, 1600 SW Archer Road, Gainesville, FL 32610, USA; ^3^Department of Urology, University of Florida Shands Cancer Center, 1600 SW Archer Road, Gainesville, FL 32610, USA

## Abstract

In the management of patients with prostate cancer, the development of new radiographic findings can mimic progression of the disease, thereby triggering changes in treatment. Typically, clinicians evaluate additional parameters, such as symptoms and prostate specific antigen (PSA) levels, for further evidence of disease progression. In the absence of additional findings, for example, elevated PSA, the possibility of an additional malignancy should be considered and evaluated. We present three cases of patients undergoing treatment for prostate adenocarcinoma and discovered on imaging to have findings suggestive of disease progression, but ultimately found to be a new primary malignancy. Our cases suggest that, in patients with prostate cancer, the appearance of new lymphadenopathy or bone lesions cannot be assumed to solely represent progression of the prostate cancer and warrant further investigation, especially in the presence of stable PSA levels.

## 1. Introduction

Prostate cancer is the second most common malignancy in men worldwide with a projected estimate for B2014 in the United States for 233,000 new cases and 29,500 deaths [1]. Metastatic prostate can present in a variety of ways, usually with a pattern of spread to the lymph nodes, regional or distant, or to the bones [2]. This case series describes three examples of patients with adenocarcinoma of the prostate with the development of radiographic evidence suggestive of progression of disease, but subsequent biopsy confirmed new primary malignancy.

## 2. Case Presentation

### 2.1. Case 1

**Figure 1:**
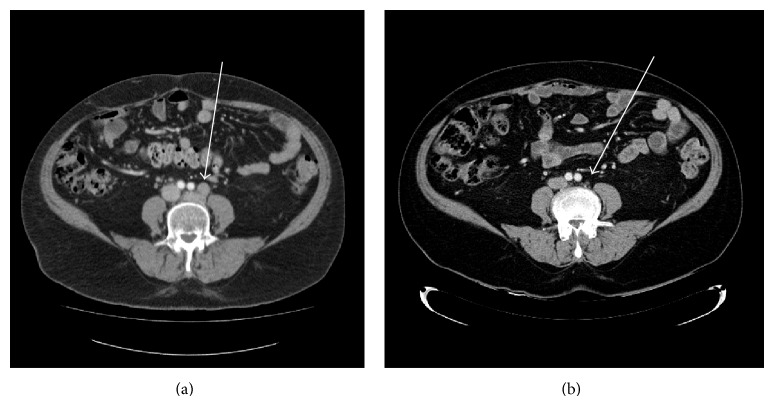
(a) Illustrated CT of the abdomen with pretreatment lymphadenopathy; (b) CT of the abdomen after chemotherapy with resolution of lymphadenopathy.

A 71-year-old Caucasian male with history of heart transplant on chronic immunosuppression and presumed metastatic prostate cancer secondary to lymphadenopathy (Gleason score: 4 + 4 = 8) on androgen deprivation therapy (ADT) with leuprolide 22.5 mg IM every 3 months underwent restaging imaging for his prostate cancer. A computerized tomography (CT) of the abdomen and pelvis, 4 months following the initiation of ADT, demonstrated enlarging pelvic and abdominal lymphadenopathy. Specifically, multiple left external iliac lymph nodes were enlarged as compared to CT from four months previously, with one lymph node enlarging from 2.7 cm × 1.7 cm to 3.0 cm × 2.0 cm and another lymph node increasing from 1.9 cm × 1.3 cm to 2.6 cm × 1.4 cm and multiple enlarging periaortic lymph nodes. Interestingly, PSA was stable, 0.4 ng/mL (normal 0–4 ng/mL) compared to 32.72 ng/mL at the time of his diagnosis. Patient denied B symptoms, that is, fevers, drenching night sweats, or weight loss >10%. A core needle biopsy of the left inguinal lymph node was compatible with follicular lymphoma, grade 3, CD20(+), and immunostain for Ki-67 shows focally increased Ki-67 positive cells and cells in the lymphoid follicles. Bone marrow biopsy demonstrated no involvement with the lymphoma. Patient was staged as stage II follicular lymphoma, grade 3. Patient completed six cycles of rituximab, cyclophosphamide, doxorubicin, vincristine, and prednisone (R-CHOP) with complete response demonstrated by CT, that is, total resolution of the abdominal and pelvic lymphadenopathy (Figure 1).

### 2.2. Case 2

**Figure 2:**
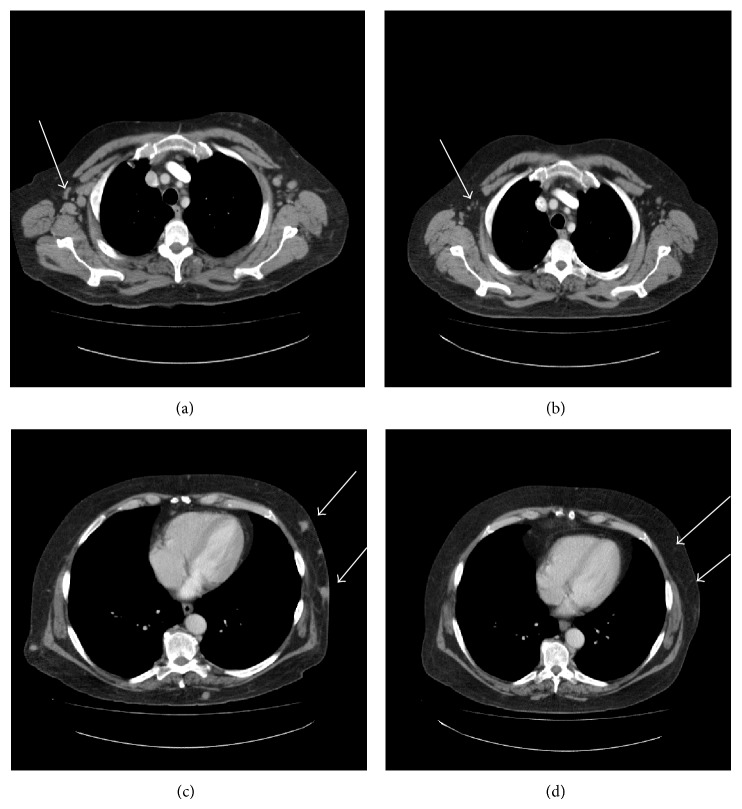
(a) Illustrated CT of the thorax with pretreatment bilateral axillary lymphadenopathy; (b) illustrated CT of the thorax with resolving bilateral axillary lymphadenopathy after the completion of chemotherapy; (c) illustrated CT of the thorax with subcutaneous nodules before treatment; (d) illustrated CT of the thorax with resolution of the subcutaneous nodules after the completion of chemotherapy.

A 67-year-old Caucasian male with history of follicular lymphoma, grade 1, in remission after four cycles of single agent rituximab, and metastatic prostate cancer on ADT with triptorelin 11.25 mg every 3 months and bicalutamide 50 mg daily was noted to have progressive lymphadenopathy on surveillance imaging. CT of the chest, abdomen, and pelvis demonstrated prominent lymph nodes in the axilla, inguinal, and periaortic regions, in addition, to multiple subcutaneous nodules. His prostate cancer was diagnosed 18 months previously having pT2cN0M1 disease with a prebiopsy PSA 50.9 ng/mL. Following ADT, his PSA nadir was 0.1 ng/mL. PSA then rises from 3.96 ng/mL to 5.12 ng/mL over two months. Patient underwent a punch biopsy of a subcutaneous nodule from his proximal left thigh and right medial elbow. The specimen from the elbow biopsy found neoplastic cells, which were medium to large in size and were CD20(+), CD30(+), CD10(+), BCL6(+), BCL2(+), CD5(−), cyclinD1(−), and CD138(−) with frequent labeling of nuclei by Ki-67. These findings were reported to be consistent with large B cell lymphoma. The specimen from the thigh biopsy demonstrated neoplastic cells found to be CD20(+), PAX5(+), and CD30(+/−) B cells with variable labeling of nuclei by Ki-67. Collectively, the findings from both biopsies were consistent with diagnosis of diffuse large B cell lymphoma. Bone marrow biopsy found no involvement with the lymphoma. Patient received six cycles of rituximab, cyclophosphamide, doxorubicin, vincristine, and prednisone (R-CHOP) and achieved a complete remission as demonstrated by CT with resolution of the generalized lymphadenopathy and subcutaneous nodules (Figure 2).

### 2.3. Case 3

**Figure 3:**
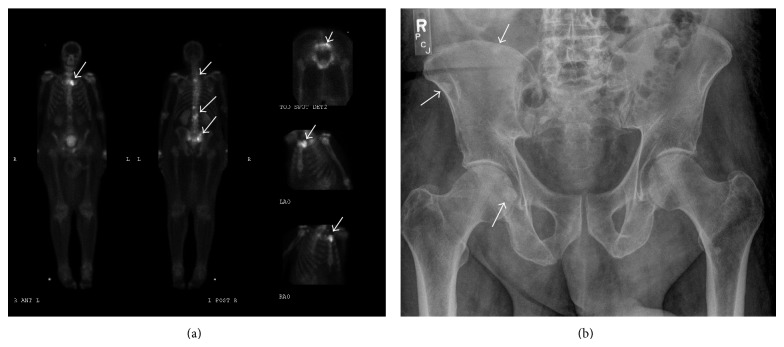
(a) Illustrated nuclear bone scan with multifocal metastatic disease; (b) illustrated pelvic X-ray with multiple metastatic bone lesions.

A 70-year-old Caucasian male with monoclonal gammopathy of undetermined significance (MGUS) and high risk adenocarcinoma of the prostate (T3b, Gleason score: 3 + 4 = 7, prebiopsy PSA: 74.3 ng/mL) who completed external beam radiation therapy to the prostate, periprostatic lymphatics, seminal vesicles, and regional pelvic lymphatics concurrent with ADT (leuprolide 22.5 mg IM every 3 months) was found to have evidence of sclerotic and blastic bony lesions, suspicious for metastatic prostatic cancer, 6 months following primary therapy with restaging imaging (Figure 3). At the time, the treatment for his adenocarcinoma of the prostate was leuprolide 22.5 mg IM every 3 months with stable PSA 1.76 ng/mL. Additionally, he was undergoing active surveillance for his MGUS and his lambda light chains were starting to increase, from 176 mg/dL to 244 mg/dL with kappa light chains 2.18 mg/dL; lambda/kappa ratio 0.01; alkaline phosphatase 84 U/L; M-spike 0.2 g/dL; IgG 538 mg/dL; IgA 26 mg/dL; and IgM <5 mg/dL. To further clarify the etiology of the bony lesions (prostate cancer versus progression of MGUS to multiple myeloma), patient underwent a CT guided biopsy of the right anterior superior iliac spine (ASIS) lesion with pathology that reported blood and fragments of hematopoietic marrow with plasma cell clusters consistent with involvement by plasma cell neoplasm. Also pathology reported an immunostain for CD138 showing increased numbers of plasma cells and clusters of plasma cells with the hematopoietic marrow, consistent with involvement by the plasma cell neoplasm, and an immunostain for cytokeratin AE1/3 was negative, excluding the possibility of metastatic carcinoma, and the presence of a plasma cell neoplasm within the marrow does not likely explain the radiographic finding of osteoblastic lesions. To further clarify the diagnosis, a rebiopsy of the right ASIS resulted in pathology reporting positive for increased CD138(+) plasma cells (20%) with findings consistent with involvement by plasma cell neoplasm and negative for evidence of metastatic carcinoma. A bone marrow biopsy was reported to demonstrate variable cellular marrow (20–40%), with multilineage hematopoiesis and maturation; clonal plasma cells comprise 15–20% of total overall marrow cellularity and they are phenotypically abnormal (CD56+/CD19−/CD45dim) and lambda restricted.

## 3. Discussion

The clinical presentation of progressive metastatic prostate cancer can manifest in metastasis to the lymph nodes, bones, liver, lungs, or brain and is typically associated with a rising PSA representing castration-recurrent disease. The development of lymphadenopathy may signify a manifestation of multiple diseases and, in particular with the elderly, triggers a differential ranging from metastatic solid tumors to hematologic malignancies, such as lymphomas [3]. In individuals with metastatic prostate cancer, newly developing lymphadenopathy can represent progression of their disease. Metastatic mechanisms of spread typically are found in the lymph nodes, by local extension or through hematogenous dissemination [4]. The manifestation of cutaneous metastasis from prostate cancer is relatively rare, accounting for 1% of all cutaneous metastasis but, nevertheless, has been reported in the literature [5, 6]. In the absence of other indications of progressive disease, that is, development of bony metastasis or rapidly increasing PSA levels, the suspicion for another neoplastic process should be high. There were no cases presented in the literature describing newly diagnosed non-Hodgkin's lymphoma in individuals undergoing active treatment for their metastatic prostate cancer. Conversely, the incidental finding of lymphoma at the time of initial diagnosis of the prostate cancer at radical prostatectomy is well documented in the literature with incidences ranging from 0.2% to 1.2% [7, 8]. Prostate cancer demonstrates a predilection for bone metastasis with incidences reported as high as 70% with the ribs, spine, and pelvis most commonly affected [9].

In PSA stable patients who have high risk or metastatic prostate cancer, bone scan and CT scan are typically done every 6–12 months as part of disease surveillance. All the three of our patients have either high risk or metastatic prostate cancer. The reason why surveillance scans are typically needed in the setting of stable PSA is that a subgroup of patients do have progression with non-PSA secreting clones of prostate cancer. These patients would then receive various treatments for metastatic castration-recurrent prostate cancer (mCRPC). Our case series shows that patients with radiologic progression with stable PSA cannot be assumed to have mCRPC and that another primary malignancy should be considered. Biopsy would then be necessary to differentiate between mCRPC and another malignancy.

## 4. Conclusion

In individuals with prostate cancer, the development of new radiographic findings can mimic progression of the disease. Our cases suggest that, in adenocarcinoma of the prostate, the appearance of new or enlarging lymphadenopathy or new bony lesions cannot be assumed to solely represent progression of the prostate cancer and warrants further investigation especially in the presence of stable PSA levels.

## Abbreviations

PSA: Prostate specific antigen
CT: Computerized tomography
ADT: Androgen deprivation therapy
R-CHOP: Rituxan, cyclophosphamide, doxorubicin, vincristine, and prednisone
MGUS: Monoclonal gammopathy of undetermined significance.
